# Cost-effectiveness of dostarlimab plus chemotherapy for primary advanced or recurrent endometrial cancer

**DOI:** 10.3389/fphar.2024.1391896

**Published:** 2024-06-20

**Authors:** Gengwei Huo, Ying Song, Wei Liu, Hua Guo, Peng Chen

**Affiliations:** ^1^ Department of Thoracic Oncology, Tianjin Medical University Cancer Institute and Hospital, Tianjin, China; ^2^ National Clinical Research Center for Cancer, Tianjin, China; ^3^ Key Laboratory of Cancer Prevention and Therapy of Tianjin, Tianjin, China; ^4^ Tianjin’s Clinical Research Center for Cancer, Tianjin, China; ^5^ Department of Pharmacy, Jining No.1 People’s Hospital, Jining, Shandong, China; ^6^ Department of Tumor Cell Biology, Tianjin Medical University Cancer Institute and Hospital, Tianjin, China

**Keywords:** dostarlimab, endometrial cancer, Markov model, RUBY, cost-effectiveness

## Abstract

**Objective:**

In the double-blind, phase III, placebo-controlled RUBY randomized clinical trial, dostarlimab plus carboplatin-paclitaxel significantly increased survival among patients with primary advanced or recurrent endometrial cancer (EC). We conducted a cost-effectiveness analysis of dostarlimab in combination with chemotherapy in these patients stratified by mismatch repair-deficient (dMMR) and mismatch repair-proficient (pMMR) subgroups from the perspective of a United States payer.

**Materials and methods:**

A Markov model with three states was employed to simulate patients who were administered either dostarlimab in combination with chemotherapy or chemotherapy based on the RUBY trial. Quality-adjusted life-years (QALYs), lifetime costs, and incremental cost-effectiveness ratio (ICER) were calculated with a willingness-to-pay (WTP) threshold of $150,000 per QALY. Both univariate and probabilistic sensitivity analyses were carried out to explore the robustness of the model.

**Results:**

In dMMR EC, the combination of dostarlimab and chemotherapy achieved an additional 5.48 QALYs at an incremental cost of $330,747 compared to chemotherapy alone, resulting in an ICER of $60,349.30 per QALY. In pMMR EC, there were 1.51 additional QALYs gained at an extra cost of $265,148, yielding an ICER of $175,788.47 per QALY. With a 15.2% discount on dostarlimab, the ICER decreased to $150,000 per QALY in the pMMR EC. The univariate sensitivity analysis revealed that the cost of dostarlimab, utility of progression-free survival (PFS), and progressive disease (PD) had the most significant impacts on the outcomes. Probabilistic sensitivity analysis revealed that dostarlimab had a 100% likelihood of being considered cost-effective for patients at a WTP threshold of $150,000 per QALY for dMMR EC, whereas this likelihood was only 0.5% for pMMR EC.

**Conclusion:**

Dostarlimab in combination with chemotherapy was cost-effective for primary advanced or recurrent dMMR EC from the perspective of a United States payer at a WTP threshold of $150,000 per QALY, but not for pMMR EC. Lowering the prices of dostarlimab could potentially enhance the cost-effectiveness of treatment for pMMR EC.

## 1 Introduction

Endometrial cancer (EC) is one of the most common types of gynecological malignancies. According to data provided by the American Cancer Society, there were roughly 66,200 new cases of EC and 13,030 deaths caused by EC in the United States in 2023 (Siegel et al., 2023). Primary advanced EC represents less than 10% of all EC diagnoses (Sung et al., 2021). While most patients are diagnosed at an early stage with localized disease, resulting in a high survival rate of 95% after 5 years, those with metastatic or recurrent disease have lower response rates to treatment and a poor prognosis. The prognosis for primary advanced EC remains unfavorable (Li et al., 2019). Among EC patients, the risk of recurrence is approximately 10%–15% for early-stage disease (Keys et al., 2004) and increases significantly to 40%–70% in advanced international federation of gynecology and obstetrics (FIGO) stages (Kurra et al., 2013). The 5-year survival rate for patients who experience a recurrence of pelvic disease is 55%, but this rate drops to 17% for individuals with recurrent disease that extends beyond the pelvic area (Xu et al., 2016).

For an extended duration, the utilization of carboplatin/paclitaxel (CP) chemotherapy as the primary treatment for individuals diagnosed with primary advanced or recurrent EC has been widely accepted as the standard approach. However, approximately 50% of patients experience disease recurrence/progression (Miller et al., 2020). Over the last 20 years, research in the field of immunobiology and the use of immune checkpoint blockade therapy for cancer treatment have significantly fueled the exploration of immunotherapy as a powerful approach for EC (Kiyotani et al., 2021; Wu, 2022; Mahdi et al., 2023).

Dostarlimab is a humanized monoclonal antibody of the lgG4 isotype. By blocking the interactions between programmed death receptor 1 (PD-1) and its ligands PD-L1 and PD-L2, dostarlimab restores the immune system’s ability to respond (Kiyotani et al., 2021; Costa and Vale, 2022). In the year 2020, a phase I clinical trial known as GARNET was conducted to evaluate the efficacy and safety of dostarlimab monotherapy in patients with mismatch repair deficient (dMMR) EC that had progressed after receiving platinum-based chemotherapy. The results of this trial demonstrated a significant enhancement in clinically relevant and long-lasting anti-tumor activity, while maintaining an acceptable safety profile (Oaknin et al., 2020). Dostarlimab was granted accelerated approval by the United States Food and Drug Administration (FDA) in April 2021 for the treatment of advanced or recurrent EC in adults with dMMR that has shown progression despite prior or ongoing treatment using platinum-containing chemotherapy.

The RUBY trial, a double-blind, phase III, placebo-controlled study (Mirza et al., 2023), demonstrated that the addition of dostarlimab to carboplatin and paclitaxel (DCP) significantly improved progression-free survival (PFS) compared to CP, especially for patients with primary advanced or recurrent dMMR EC. Furthermore, the study indicated a beneficial trend in PFS and overall survival (OS) for patients with mismatch repair-proficient (pMMR) EC. Based on these findings, the FDA granted approval on 31 July 2023, for the use of DCP therapy, as well as sequential monotherapy with dostarlimab, as treatment options for primary advanced or recurrent dMMR/microsatellite instability high (MSI-H) EC.

Given the significant benefit seen in dMMR EC, as well as the National Comprehensive Cancer Network (NCCN)’s recognition of pMMR EC as a potential subgroup that could benefit from treatment, dostarlimab is beginning to be used both for dMMR and pMMR EC patients in clinical practice. However, considering its comparatively high price, it is imperative to evaluate the cost-effectiveness attributes of dostarlimab in these two subgroups of patients. From the standpoint of United States payers, our investigation sought to assess the cost-effectiveness of DCP therapy in comparison to CP therapy for patients with primary advanced or recurrent EC.

## 2 Methods

### 2.1 Model construction

The TreeAge Pro 2022 software (TreeAge, Massachusetts, United States) was used to construct a Markov model. Subsequently, statistical analysis was conducted using R software (version 4.2.1). The model framework encompasses three distinct and mutually exclusive health states: PFS, progressive disease (PD), and death. These health states are illustrated in Supplementary Figure S1.

Based on the RUBY trial, patients with primary advanced or recurrent EC, who had a median age of 64, were stratified into dMMR and pMMR subgroups (Mirza et al., 2023). Both dMMR and pMMR subgroups were further subdivided into two distinct treatment groups: an experimental group and a control group. The experimental group initially received dostarlimab (500 mg) combined with carboplatin (AUC = 5) and paclitaxel (175 mg/m^2^) intravenously every 3 weeks for six cycles. This was followed by dostarlimab (1,000 mg) intravenously every 6 weeks for a duration of up to 3 years, constituting the DCP group. The control group, designated as the CP group, was initially treated with carboplatin (AUC = 5) and paclitaxel (175 mg/m^2^) intravenously every 3 weeks for six cycles, followed by regular follow-up.

Following the RUBY trial protocol (Mirza et al., 2023), subsequent anticancer therapeutics were administered to 28.3% of patients in the DCP group and 58.5% of patients in the CP group upon disease progression. The remaining patients received the best supportive care. However, due to the RUBY trial’s lack of detailed descriptions concerning treatment regimens following disease progression, and based on common clinical practice, we postulate that each group likely received either single-agent chemotherapy (doxorubicin 60 mg/m^2^ administered every 3 weeks for six cycles) or immunotherapy (pembrolizumab 200 mg given every 3 weeks), or, alternatively, continued with the best supportive care (Supplementary Table S1).

Every 3 weeks constituted a model cycle. The primary outcomes of our analysis encompassed overall costs, quality-adjusted life-years (QALYs), and incremental cost-effectiveness ratios (ICERs). Half-cycle correction and a 3% annual discount rate were used in the calculation of costs and life expectancy (Liu et al., 2022).

### 2.2 Costs estimates

The evaluation of costs was carried out from the perspective of third-party payers in the United States We considered health resource utilization and direct medical expenses, which encompassed drug procurement, disease management, drug administration, and treatment-related adverse events (Table 1). The drug dosage was determined based on an average American woman’s body surface area of 1.84 m^2^ (Liu et al., 2022).

**TABLE 1:** Model parameters and distributions.

Variable	Baseline value	Range		Distribution	References
		Minimum	Maximum		
Gen-gamma PFS survival model with dMMR DCP group	mu = 1.42499 sigma = 1.58999 Q = −3.37074	-	-	-	-
Log-logistic PFS survival model with dMMR CP group	shape = 2.05127; scale = 8.08222	-	-	-	-
Gen-gamma OS survival model with dMMR DCP group	mu = 0.32405 sigma = 0.58157 Q = −33.94592	-	-	-	-
Log-normal OS survival model with dMMR CP group	meanlog = 3.56218 sdlog = 1.33010	-	-	-	-
Log-normal PFS survival model with pMMR DCP group	meanlog = 2.49712 sdlog = 1.07087	-	-	-	-
Log-logistic PFS survival model with pMMR CP group	shape = 1.87448 scale = 9.19005	-	-	-	-
Log-normal OS survival model with pMMR DCP group	meanlog = 3.77032 sdlog = 1.33401	-	-	-	-
Log-logistic OS survival model with pMMR CP group	shape = 1.78643 scale = 27.54835	-	-	-	-
Grade ≥3 AEs incidence in DCP group					
Anemia	0.149	0.1192	0.1788	Beta	Mirza et al. (2023)
Neutropenia	0.095	0.076	0.114	Beta	Mirza et al. (2023)
Neutrophil count decreased	0.083	0.0664	0.0996	Beta	Mirza et al. (2023)
Lymphocyte count decreased	0.054	0.0432	0.0648	Beta	Mirza et al. (2023)
White-cell count decreased	0.066	0.0528	0.0792	Beta	Mirza et al. (2023)
Hypertension	0.071	0.0568	0.0852	Beta	Mirza et al. (2023)
Pulmonary embolism	0.050	0.04	0.06	Beta	Mirza et al. (2023)
Hypokalemia	0.050	0.04	0.06	Beta	Mirza et al. (2023)
Grade ≥3 AEs incidence in CP group					
Anemia	0.163	0.1304	0.1956	Beta	Mirza et al. (2023)
Neutropenia	0.093	0.0744	0.1116	Beta	Mirza et al. (2023)
Neutrophil count decreased	0.138	0.1104	0.1656	Beta	Mirza et al. (2023)
Lymphocyte count decreased	0.073	0.0584	0.0876	Beta	Mirza et al. (2023)
White-cell count decreased	0.053	0.0424	0.0636	Beta	Mirza et al. (2023)
Hypertension	0.033	0.0264	0.0396	Beta	Mirza et al. (2023)
Pulmonary embolism	0.049	0.0392	0.0588	Beta	Mirza et al. (2023)
Hypokalemia	0.037	0.0296	0.0444	Beta	Mirza et al. (2023)
AEs cost, U.S.$					
Anemia	14,757.94	11,806.35	17,709.53	Gamma	Feng et al. (2022)
Neutropenia	14,809.28	11,847.42	17,771.14	Gamma	Thurgar et al. (2021)
Neutrophil count decreased	14,809.28	11,847.42	17,771.14	Gamma	Thurgar et al. (2021)
Lymphocyte count decreased	7,257.66	5,806.13	8,709.19	Gamma	Thurgar et al. (2021)
White-cell count decreased	7,257.66	5,806.13	8,709.19	Gamma	Thurgar et al. (2021)
Hypertension	8,212.53	6,570.02	9,855.04	Gamma	Feng et al. (2022)
Pulmonary embolism	87,717.73	70,174.18	105,261.28	Gamma	Thurgar et al. (2021)
Hypokalemia	7,332.99	5,866.39	8,799.59	Gamma	Kuznik et al. (2022)
AEs disutility					
Anemia	0.073	0.0584	0.0876	Beta	Liu et al. (2022)
Neutropenia	0.09	0.072	0.108	Beta	Nafees et al. (2008)
Neutrophil count decreased	0.09	0.072	0.108	Beta	Nafees et al. (2008)
Lymphocyte count decreased	0.09	0.072	0.108	Beta	Nafees et al. (2008)
White-cell count decreased	0.09	0.072	0.108	Beta	Nafees et al. (2008)
Hypertension	0.05	0.04	0.06	Beta	Nafees et al. (2017)
Pulmonary embolism	0.10	0.08	0.12	Beta	estimated
Hypokalemia	0.05	0.04	0.06	Beta	estimated
Utility					
Progression-free survival	0.817	0.6536	0.9804	Beta	Thurgar et al. (2021)
Progressed disease	0.779	0.6232	0.9348	Beta	Thurgar et al. (2021)
Drug cost, U.S.$					
Dostarlimab/10 mg	233.258	186.61	279.91	Fixed in PSA	CMM (2024)
Pembrolizumab/1 mg	55.730	44.58	66.88	Fixed in PSA	CMM (2024)
Doxorubicin/10 mg	3.279	2.62	3.93	Gamma	CMM (2024)
Paclitaxel/1 mg	0.108	0.09	0.13	Gamma	CMM (2024)
Carboplatin/50 mg	3.599	2.88	4.32	Gamma	CMM (2024)
Tumor imaging cost per cycle	651.81	521.45	782.17	Gamma	Barrington et al. (2020)
Laboratory testing cost per cycle	359.16	287.33	430.99	Gamma	Ding et al. (2021)
Patients’ body surface area, m^2^	1.84	1.47	2.21	Normal	Liu et al. (2022)
Administration cost per cycle	149.69	119.75	179.63	Gamma	Liu et al. (2022)
Physician visit cost per cycle	164.04	131.23	196.85	Gamma	CDC (2023)
End-of-life care in end-stage disease one-time cost	37,590.23	30,072.18	45,108.28	Gamma	Ackroyd et al. (2021)
Best supportive care per cycle	1,300.61	1,040.49	1,560.73	Gamma	Liu et al. (2022)
Discount rate (%)	3	-	-	-	Liu et al. (2022)

AEs, adverse effects; CP, carboplatin and paclitaxel; DCP, dostarlimab combined with carboplatin and paclitaxel; OS, overall survival; PFS, progression-free survival.

We extracted drug prices from the Centers for Medicare and Medicaid Services (CMM, 2024). The expenses associated with the administration of medication, best supportive care, end-of-life palliative care, and disease management (which includes costs related to physician visits, computed tomography, and laboratory examinations) were obtained from previously published pre-existing databases (Barrington et al., 2020; Ackroyd et al., 2021; Ding et al., 2021; Feng et al., 2022; Kuznik et al., 2022; Liu et al., 2022; CDC, 2023). Based on the RUBY trial and clinical practice (Mirza et al., 2023), computed tomography scans were performed at 6-week intervals starting from the initial treatment until week 25, then at 9-week intervals until week 52, and subsequently every 12 weeks until the presence of progressive disease. Laboratory testing and administration costs were documented in every treatment cycle.

To account for inflation and adjust the costs to 2024 United States dollar values, we used the American Consumer Price Index (CPI) for cost adjustments. Specifically, we utilized the Tom’s Inflation Calculator to update the costs to 2024 levels (Halfhill, 2024). Additionally, we applied a willingness-to-pay (WTP) threshold of $150,000 per QALY in our analysis of cost-effectiveness (Ackroyd et al., 2021).

### 2.3 Survival and progression transition estimates

The transition probabilities based on the PFS and OS curves from the RUBY trial were extrapolated using the GetData Graph Digitizer software (version 2.22). The algorithm developed by Hoyle et al. was employed to generate simulated patient data (Hoyle and Henley, 2011). These extracted data points were then fitted to various survival functions, including exponential, log-logistic, log-normal, gamma, Weibull, and Gompertz, among others. The optimal fit was determined using the Akaike and Bayesian Information Criteria (Supplementary Figure S2; Supplementary Tables S2, S3). We employed the concept of the area under the PFS curve to represent the cumulative patient population in the pre-progression health state over time. Similarly, the area above the OS curve indicated the cumulative patient population in the deceased health state. The area between these curves represented the cumulative patient population in the post-progression health state. Using Microsoft Excel Software, we computed time-dependent transition probabilities for both patient groups, incorporating data from the RUBY trial. These probabilities were then extrapolated to cover a lifetime horizon. The formula for calculating transition probability values in each model cycle is as follows: transition probabilities (tu) = 1 - exp [λ(t - u)^γ^ - λt^γ^], where λ > 0 and γ > 0. In this formula, “u” denotes the model cycle, while “tu” signifies the transition to state “t” after “u” cycles. The model’s timeframe was set between 64 and 82 years of age to align with the median age of participants in the RUBY trial and the average lifespan of United States females at birth (Supplementary Table S4) (Arias and Xu, 2022).

### 2.4 Health-state utilities

The health utility values for PFS, PD, death, and adverse effects (AEs) were derived from previously published investigations (Nafees et al., 2008; 2017; Thurgar et al., 2021). Consistent with traditional research methods, the main focus is on severe AEs (grade ≥3) that occur at an incidence rate of 5% or higher (You et al., 2023). This is primarily because mild AEs usually do not require treatment or incur substantial treatment costs (Thurgar et al., 2021). The reduction in QALYs associated with AEs was factored into the models’ initial cycle (Su et al., 2021). All parameters related to the utilities are presented in Table 1.

### 2.5 Univariate and probabilistic sensitivity analyses

We systematically adjusted clinical parameters within a range that accounted for plausible deviations of 20% from their baseline values in the univariate sensitivity analysis. These corresponding variations are visually presented in the tornado diagram. We employed 1,000 Monte Carlo simulations to perform a sensitivity analysis on the probability. This involved simultaneously and randomly varying preset parameters according to specific distribution patterns. The costs follow a gamma distribution, while the proportion and utility follow beta distributions (Table 1).

## 3 Results

### 3.1 Base case results

In the dMMR group, the cumulative costs totaled $685,620 for the DCP group and $354,873 for the CP group. The DCP group resulted in 8.97 QALYs, while the CP group yielded 3.49 QALYs. As a result, individuals in the DCP group gained an increase of 5.48 QALYs with an additional cost of $330,747 compared to the CP group. This led to an ICER of $60,349.30/QALY, within the predetermined WTP threshold of $150,000/QALY (Table 2).

**TABLE 2:** Base-case results (cost, QALY, and ICER) of the model.

Group	Costs, U.S. $	△Costs, U.S. $	QALYs	△QALYs	ICER U.S. $/QALY
dMMR CP	354,873	–	3.49	–	–
dMMR DCP	685,620	330,747	8.97	5.48	60,349.30
pMMR CP	228,311	–	2.48	–	–
pMMR DCP	493,458	265,148	3.99	1.51	175,788.47

CP, carboplatin and paclitaxel; DCP, dostarlimab combined with carboplatin and paclitaxel; ICER, incremental cost-effectiveness ratio; QALYs, quality-adjusted life-years.

In the pMMR group, the cumulative costs amounted to $493,458 for the DCP group and $228,311 for the CP group. The DCP group resulted in 3.99 QALYs, while the CP group yielded 2.48 QALYs. Consequently, individuals in the DCP group experienced an increase of 1.51 QALYs at an extra cost of $265,148 compared to the CP group. This resulted in an ICER of $175,788.47/QALY, surpassing the predetermined WTP threshold of $150,000/QALY (Table 2).

### 3.2 Sensitivity analysis

**FIGURE 1:**
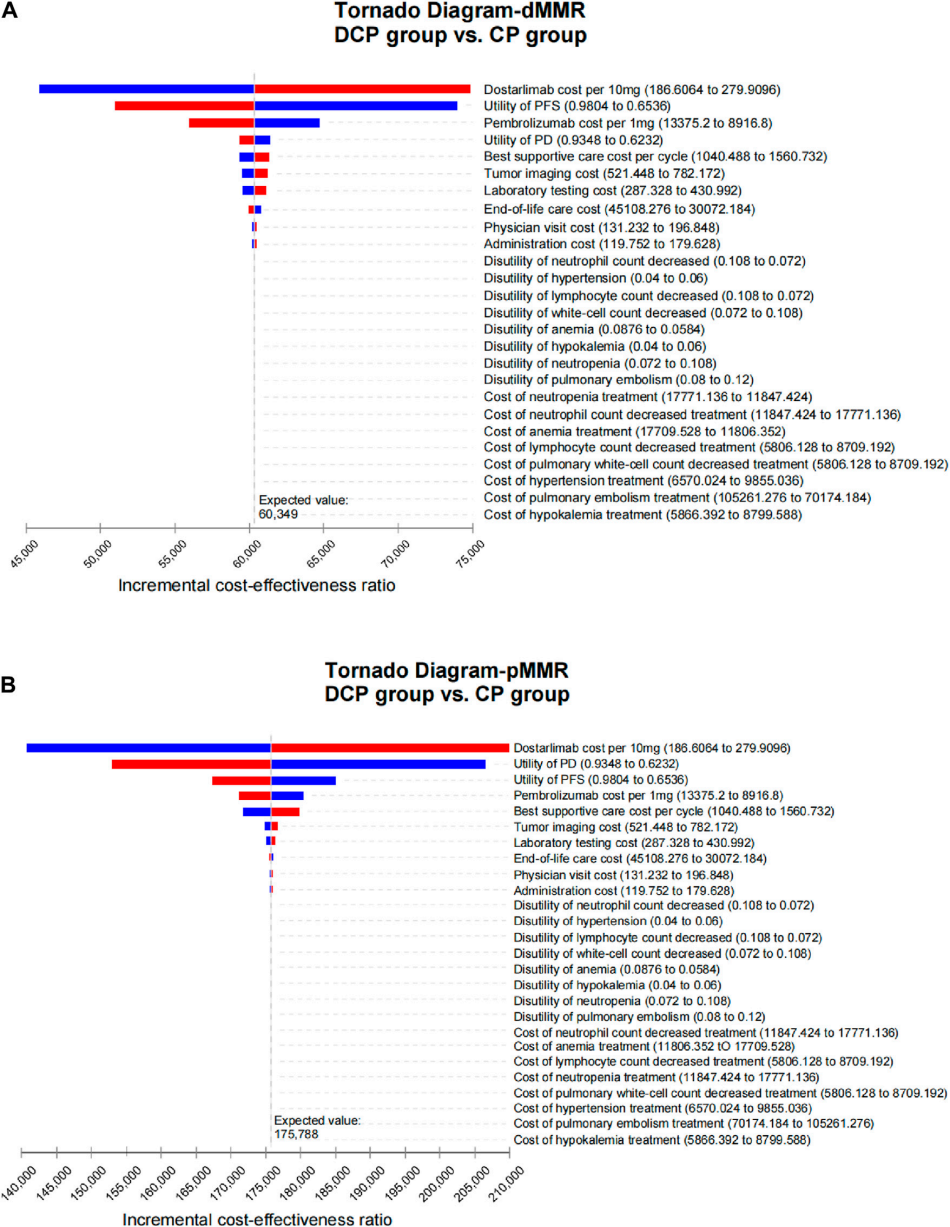
Tornado diagram illustrating the results of univariate sensitivity analyses for dMMR EC **(A)** and pMMR EC **(B)**.

As illustrated in Figure 1, the tornado diagram reveals the significant influence of specific parameters on the ICER, including the cost of dostarlimab, the utility associated with PFS and PD, and the cost of pembrolizumab. Other variables exert a minimal impact on the outcome. When dostarlimab receives a 15.2% discount, the resulting ICER drops to $150,000/QALY in the pMMR group. While other parameters vary within their respective ranges, no intersection is observed between the ICER and the predetermined WTP values.

**FIGURE 2:**
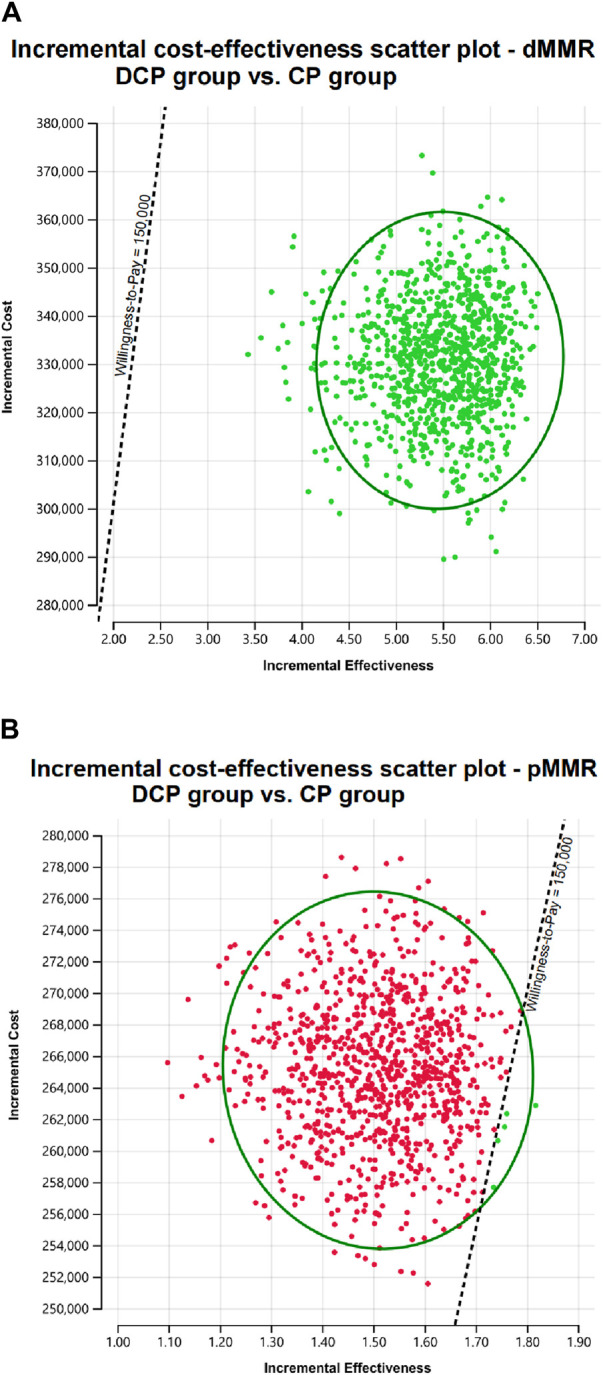
Scatter plot diagrams showing the incremental cost-effectiveness of dostarlimab plus chemotherapy compared to chemotherapy alone in dMMR EC **(A)** and pMMR EC **(B)**.

A Monte Carlo simulation indicated that all scatter points were located in the first quadrant of the coordinate plane, which suggests a higher number of QALYs gained with a higher cost. In the dMMR group, all scatter points fell below the WTP line, whereas in the pMMR group, only 0.5% of the scatter points were positioned below this line (Figure 2).

**FIGURE 3:**
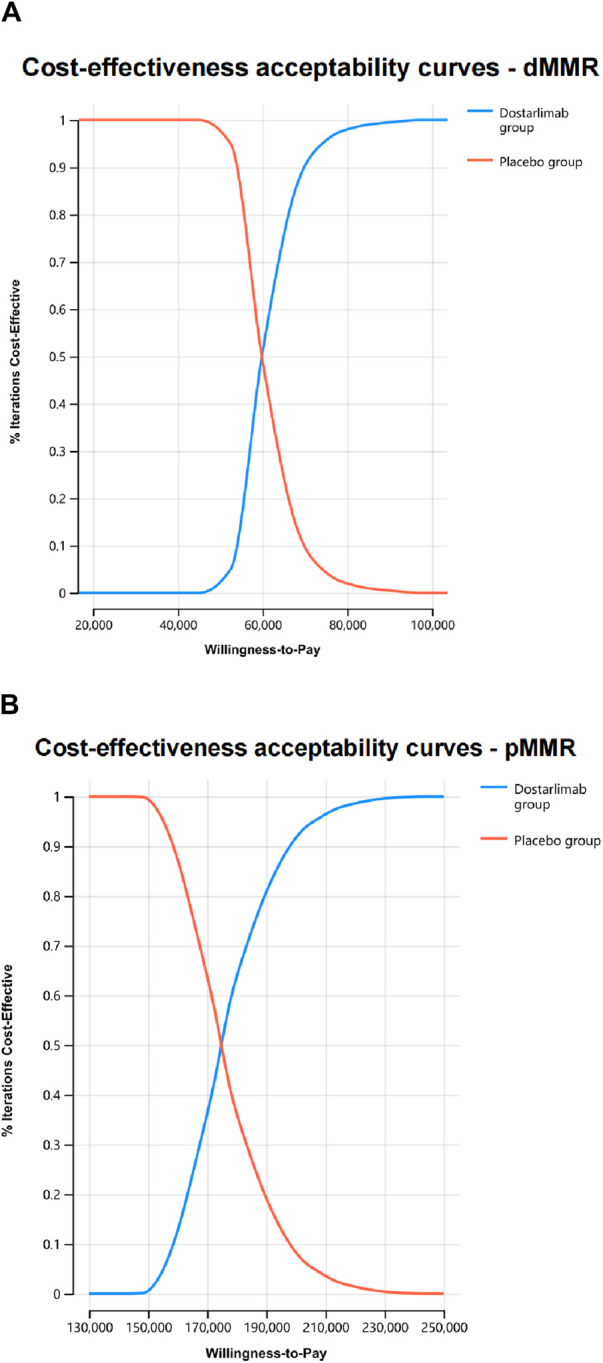
The cost-effectiveness acceptability curves were generated through probabilistic sensitivity analyses for dMMR EC **(A)** and pMMR EC **(B)**.

Probability sensitivity analysis revealed that dostarlimab had a 100% chance of being deemed cost-effective for dMMR EC. However, this likelihood was significantly lower, at only 0.5%, for pMMR EC (Figure 3).

## 4 Discussion

According to our model findings, DCP therapy demonstrates superior health outcomes compared with CP therapy (8.97 QALYs versus 3.49 QALYs in dMMR EC and 3.99 QALYs versus 2.48 QALYs in pMMR EC). However, cost-effectiveness varies between the two groups, with an ICER of $60,349.30/QALY for dMMR EC and $175,788.47/QALY for pMMR EC. The probability sensitivity analysis results indicate that while DCP therapy is considered a cost-effective treatment for dMMR EC, only 0.5% of cases in pMMR EC are deemed cost-effective. When dostarlimab receives a 15.2% discount, the resulting ICER drops to $150,000/QALY in the pMMR group.

A prior study that used a decision model evaluated the cost-effectiveness of dostarlimab monotherapy for recurrent dMMR EC (Dioun et al., 2023). This cost-effectiveness analysis was based on the phase I, single-group GARNET trials. They found that dostarlimab monotherapy is associated with greater survival compared with pembrolizumab or pegylated liposomal doxorubicin but surpasses the WTP threshold of $150,000 per QALY for recurrent dMMR EC. In contrast, our study revealed that the DCP therapy demonstrated enhanced clinical outcomes and fell below the WTP threshold of $150,000 per QALY, compared to the use of CP therapy. This finding underscores the excellent cost-effectiveness of DCP therapy in the context of this medical intervention. Due to the nature of the GARNET trials as a phase I study, only the objective response rate was reported, and there was a lack of available OS and PFS data. As a result, the model used fixed disease transition probabilities, which may not accurately reflect the natural progression of tumor-related diseases. The release of OS and PFS results from the RUBY trial has provided additional data that can be used to further investigate the cost-effectiveness profile of DCP therapy. The superior survival status of the combined therapy regimen, which has shown a 72% lower risk of progression or death in the dMMR-MSI-H population, makes this regimen highly cost-effective. The cost-effectiveness of a combined treatment approach not only alleviates the financial burden on patients and their families but also yields favorable implications for healthcare systems and insurers.

Another study evaluated the cost-effectiveness of DCP from the perspective of China’s healthcare system. They found that the ICERs were $53,063.61 per QALY for the dMMR subgroup and $124,088.56 per QALY for the pMMR subgroup. Both values exceeded China’s WTP threshold of $38,201 per QALY. Therefore, they concluded that DCP is unlikely to be a cost-effective option for advanced dMMR and pMMR EC in China (You et al., 2023). This study exhibits a similar trend to our research findings in terms of the ICER value, suggesting superior cost-effectiveness of DCP in the dMMR subgroup compared to the pMMR subgroup. However, when considering the cost-effectiveness of DCP from the United States and Chinese perspectives, a notable difference emerges. Specifically, DCP therapy was deemed cost-effective for dMMR EC in the United States, but not in China. In the United States, a developed nation with significantly higher *per capita* gross domestic product (GDP) and health expenditure, patients have a higher WTP threshold, resulting in more favorable economic outcomes for DCP. Conversely, China, being a developing country with lower *per capita* GDP and health expenditure, faces challenges regarding patients’ ability to afford medications, leading to a lower WTP threshold. National differences, including policies, economic conditions, and cultural factors, significantly influence the landscape of drug cost-effectiveness. These disparities have a direct impact on patients’ access to and affordability of medications in different countries, ultimately shaping healthcare outcomes and the overall burden on healthcare systems.

To our knowledge, this article is the first cost-effectiveness comparison study of dostarlimab in EC using dMMR and pMMR as subgroups from a United States perspective. Our model incorporated several influential factors, including the cost of dostarlimab, the utility related to PFS and PD, and the cost of optimal supportive care. The current price of dostarlimab indicates cost-effectiveness for dMMR EC. However, for dostarlimab to be deemed cost-effective for pMMR EC, a 15.2% discount would be required. The health utility data, which were used to evaluate the utility of PFS and PD statuses, were obtained from published studies specifically focused on EC patients (Thurgar et al., 2021). Using both the upper and lower bounds of utility values in sensitivity analyses did not change the overall conclusion.

This study has several limitations. Firstly, while it is essential to extend the survival curve to capture comprehensive survival outcomes within our analytical framework, the reconstructed survival curves exhibited some discrepancies with the actual data. Nonetheless, our objective in adjusting the transition probability was to closely approximate the real-world results. Secondly, in alignment with most previous studies, we focused exclusively on AEs of grade ≥3 and with an occurrence rate of ≥5% (You et al., 2023). This approach may potentially lead to an underestimation of the ICER. However, it is worth noting that the treatment costs and disutility associated with low-grade and low-frequency AEs have minimal impact on the overall outcomes. Thirdly, treatment decisions were constrained in the PD status due to variations in clinical practice. Specifically, local lesion radiotherapy, surgeries, or other treatment modalities were not considered, which may limit the real-world applicability for individuals in this state. Importantly, these considerations have minimal impact on the ICER findings. Despite these limitations, our study offers valuable insights into the cost-effectiveness of DCP therapy for primary advanced or recurrent EC from the perspective of United States payers. Future research should aim to address these limitations and further assess the long-term cost-effectiveness of dostarlimab.

## 5 Conclusion

Dostarlimab in combination with chemotherapy was cost-effective compared to chemotherapy alone for primary advanced or recurrent dMMR EC from the perspective of a United States payer at a WTP threshold of $150,000 per QALY, but not for pMMR EC. Lowering the prices of dostarlimab could potentially enhance the cost-effectiveness of treatment for pMMR EC. It is desired that additional real-world studies on dostarlimab and assessments of health outcomes will be carried out in the future, thus providing further guidance for physicians, patients, and health insurance policymakers.

## Data Availability

The original contributions presented in the study are included in the article/Supplementary Material, further inquiries can be directed to the corresponding authors.
