# Supplementary material for: Cost-effectiveness of dostarlimab plus chemotherapy for primary advanced or recurrent endometrial cancer
Source: Front Pharmacol. 2024 Jun 20;15:1391896. doi: 10.3389/fphar.2024.1391896 (PMC11222638; doi:10.3389/fphar.2024.1391896)
Supplement: Supplementary file 1 [file Table1.DOCX]

**Table S1 Treatment regimen selection for patients following disease progression.**

| Treatment regimen | dMMR | | pMMR | |
| --- | --- | --- | --- | --- |
|  | DCP group | CP group | DCP group | CP group |
| Pembrolizumab | 0.151 | 0.385 | 0.156 | 0.332 |
| Doxorubicin | 0.132 | 0.200 | 0.365 | 0.348 |
| Best supportive care | 0.717 | 0.415 | 0.479 | 0.320 |

CP: carboplatin and paclitaxel; DCP: dostarlimab combined with carboplatin and paclitaxel.

**Table S2 Akaike information criterion and Bayesian information criterion statistics for alternate parametric survival distributions in dMMR endometrial cancer.**

| Distribution | PFS | | | | OS | | | |
| --- | --- | --- | --- | --- | --- | --- | --- | --- |
|  | DCP group | | CP group | | DCP group | | CP group | |
|  | AIC | BIC | AIC | BIC | AIC | BIC | AIC | BIC |
| Exponential | 183.8041 | 185.7744 | 333.3593 | 335.5337 | 87.0473 | 89.0175 | 237.9531 | 240.1275 |
| Gamma | 184.0044 | 187.9450 | 328.8341 | 333.1828 | 88.3391 | 92.2797 | 238.8545 | 243.2033 |
| Gengamma | 174.9823 | 180.8932 | 319.2696 | 325.7927 | 86.9492 | 92.8601 | 239.9137 | 246.4368 |
| Gompertz | 175.0394 | 178.9800 | 335.1761 | 339.5249 | 87.8766 | 91.8171 | 239.8056 | 244.1544 |
| Weibull | 183.2705 | 187.2111 | 332.0707 | 336.4195 | 88.2870 | 92.2276 | 239.0500 | 243.3988 |
| Log-logistic | 181.2686 | 185.2091 | 318.1701 | 322.5189 | 88.1980 | 92.1386 | 238.4534 | 242.8022 |
| Log-normal | 179.0552 | 182.9958 | 319.4122 | 323.7610 | 87.6258 | 91.5663 | 237.9236 | 242.2724 |

AIC, Akaike information criterion; BIC: Bayesian information criterion; CP: carboplatin and paclitaxel; DCP: dostarlimab combined with carboplatin and paclitaxel.

**Table S3 Akaike information criterion and Bayesian information criterion statistics for alternate parametric survival distributions in pMMR endometrial cancer.**

| Distribution | PFS | | | | OS | | | |
| --- | --- | --- | --- | --- | --- | --- | --- | --- |
|  | DCP group | | CP group | | DCP group | | CP group | |
|  | AIC | BIC | AIC | BIC | AIC | BIC | AIC | BIC |
| Exponential | 914.1740 | 917.4315 | 957.9261 | 961.1411 | 596.5873 | 599.8448 | 732.6250 | 735.8399 |
| Gamma | 909.2196 | 915.7346 | 946.1031 | 952.5330 | 594.8294 | 601.3444 | 719.1359 | 725.5658 |
| Gengamma | 896.3425 | 906.1150 | 927.9089 | 937.5537 | 594.9201 | 604.6926 | 720.2473 | 729.8921 |
| Gompertz | 916.0586 | 922.5736 | 959.8513 | 966.2812 | 597.7340 | 604.2490 | 728.1297 | 734.5595 |
| Weibull | 912.2698 | 918.7848 | 952.2232 | 958.6531 | 595.3569 | 601.8719 | 720.6401 | 727.0700 |
| Log-logistic | 897.2613 | 903.7762 | 925.6176 | 932.0474 | 594.0698 | 600.5848 | 717.4707 | 723.9006 |
| Log-normal | 895.4418 | 901.9568 | 927.5334 | 933.9632 | 592.9232 | 599.4382 | 719.4974 | 725.9272 |

AIC, Akaike information criterion; BIC: Bayesian information criterion; CP: carboplatin and paclitaxel; DCP: dostarlimab combined with carboplatin and paclitaxel.

**Table S4 Background mortality rate.**

| **Age** | **Background mortality rate** | **Age** | **Background mortality rate** | **Age** | **Background mortality rate** |
| --- | --- | --- | --- | --- | --- |
| 60 | 0.006891 | 74 | 0.021198 | 88 | 0.101682 |
| 61 | 0.007455 | 75 | 0.023549 | 89 | 0.114054 |
| 62 | 0.008004 | 76 | 0.025917 | 90 | 0.127590 |
| 63 | 0.008520 | 77 | 0.029002 | 91 | 0.142319 |
| 64 | 0.009033 | 78 | 0.032220 | 92 | 0.158250 |
| 65 | 0.009573 | 79 | 0.035944 | 93 | 0.175367 |
| 66 | 0.010236 | 80 | 0.040043 | 94 | 0.193631 |
| 67 | 0.011030 | 81 | 0.044578 | 95 | 0.212972 |
| 68 | 0.012018 | 82 | 0.050431 | 96 | 0.233293 |
| 69 | 0.013187 | 83 | 0.056908 | 97 | 0.254465 |
| 70 | 0.014484 | 84 | 0.063205 | 98 | 0.276333 |
| 71 | 0.015956 | 85 | 0.071076 | 99 | 0.298719 |
| 72 | 0.017382 | 86 | 0.080252 | 100 | 1 |
| 73 | 0.019431 | 87 | 0.090433 |  |  |
