# Supplementary material for: Cost-effectiveness of dostarlimab plus chemotherapy for primary advanced or recurrent endometrial cancer
Source: Front Pharmacol. 2024 Jun 20;15:1391896. doi: 10.3389/fphar.2024.1391896 (PMC11222638; doi:10.3389/fphar.2024.1391896)
Supplement: Supplementary file 2 [file DataSheet1.PDF]

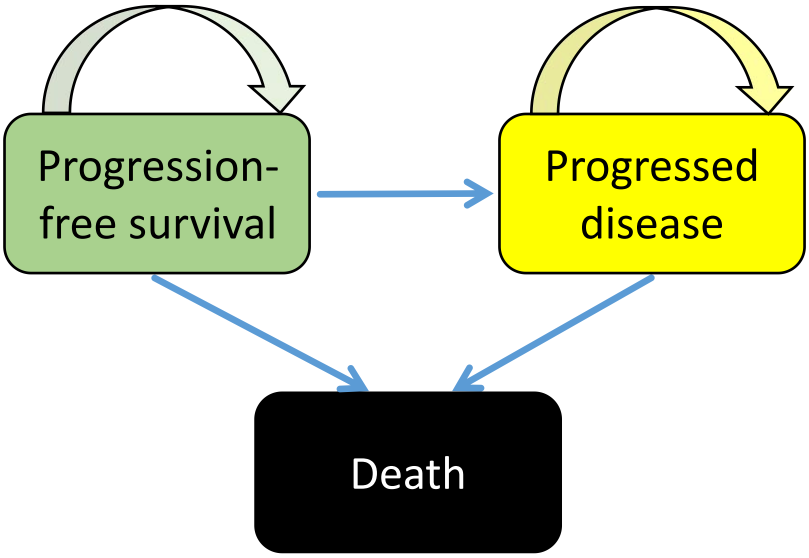

Figure S1 The Markov model simulated with three health states: progression-free survival,progressed disease and death

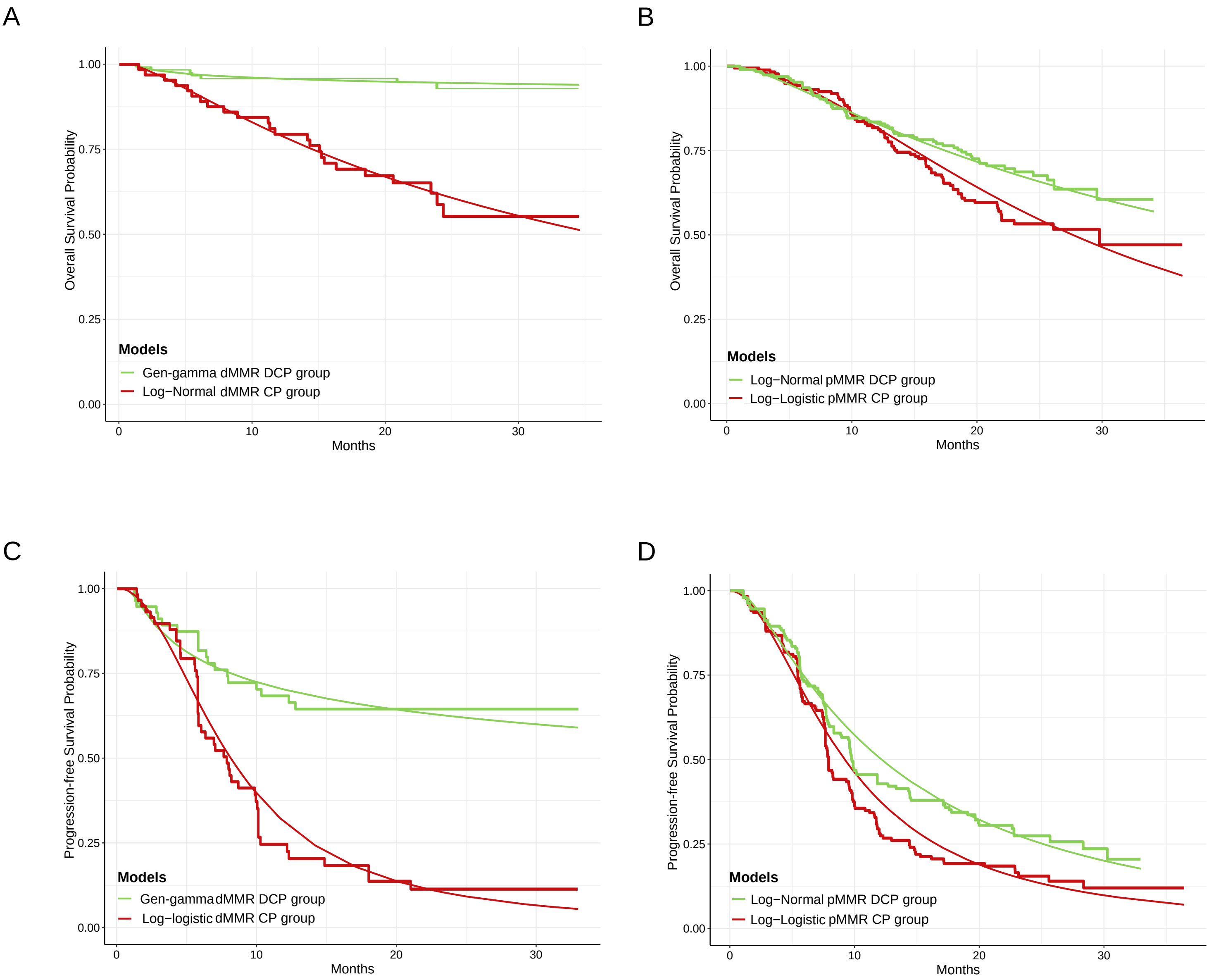

Figure S2 Estimated best-fitting overall survival curves for dMMR EC (A), pMMR EC (B). Estimated best-fitting progression-free survival curves for dMMR EC (C), pMMR EC (D).
